# Mondor's Disease of the Arm Following Breast Cancer Treatment

**DOI:** 10.7759/cureus.13421

**Published:** 2021-02-18

**Authors:** Sherif Monib, Kelvin Chong

**Affiliations:** 1 Breast Surgery, West Hertfordshire Hospitals NHS Trust, St. Albans, GBR

**Keywords:** mondor's disease, cord-like structure, breast cancer

## Abstract

Mondor's disease is a rare, peculiar form of superficial thrombophlebitis which mainly affects the subcutaneous veins of the breast, anterior chest wall, neck, axilla, upper limbs and penis.

In most cases, it presents with rapid development of a painful subcutaneous cord-like structure that later becomes less painful, but a fibrous band persists. Unfortunately, aetiology and management are not very clear, but it is a self-limiting condition in most cases.

We are presenting a rare case of a patient who developed Mondor's disease in the antecubital fossa of the right arm following chemotherapy for breast cancer.

## Introduction

Mondor's disease is a rare, peculiar form of superficial thrombophlebitis which mainly affects the subcutaneous veins of the breast, anterior chest wall, and rarely other parts including cervical region, axilla, upper limbs or the penis.

While Faage was the first one to describe this condition in 1869, it is named after Henri Mondor, a French surgeon who gave this condition further characterization and described it as a syndrome of sclerosing superficial thrombophlebitis of the veins of the anterior thoracic wall in 1939 [1].

The differential diagnosis of Mondor's disease, in general, varies according to the location of symptoms. In our case, the differential diagnosis included thrombophlebitis migrans, Buerger disease, Behçet disease, polyarteritis nodosa, cellulitis or abscess formation.

Due to the self-limited nature of the condition, there are only around 400 reported cases of Mondor's disease in medical literature [2]. There has been no correlation between race, ethnicity or age and incidence of Mondor's disease. In Amano's review, 45% of cases are idiopathic, 20% iatrogenic, 22% traumatic, and 5% were related to breast cancer [3].

## Case presentation

We present a case of a 36-year-old lady who was diagnosed with right breast cancer. She underwent a right therapeutic mammoplasty and sentinel lymph node biopsy as well as excision of bilateral accessory axillary breast tissue. The final postoperative histology showed a 30 mm grade III invasive ductal carcinoma, estrogen receptor (ER) 8, progesterone receptor (PR) 8, human epidermal growth factor receptor 2 (HER2) negative and sentinel node biopsy revealed a single non-cancerous lymph node (0/1) and her Nottingham Prognostic Index was 4.6. Her Oncotype Dx test results showed a high recurrence score, and she subsequently underwent adjuvant chemotherapy (Epirubicin, Cyclophosphamide, and Taxol). She had one episode of neutropenia after her 2nd cycle, which caused a delay in receiving her remaining four cycles of chemotherapy.

**Figure 1:**
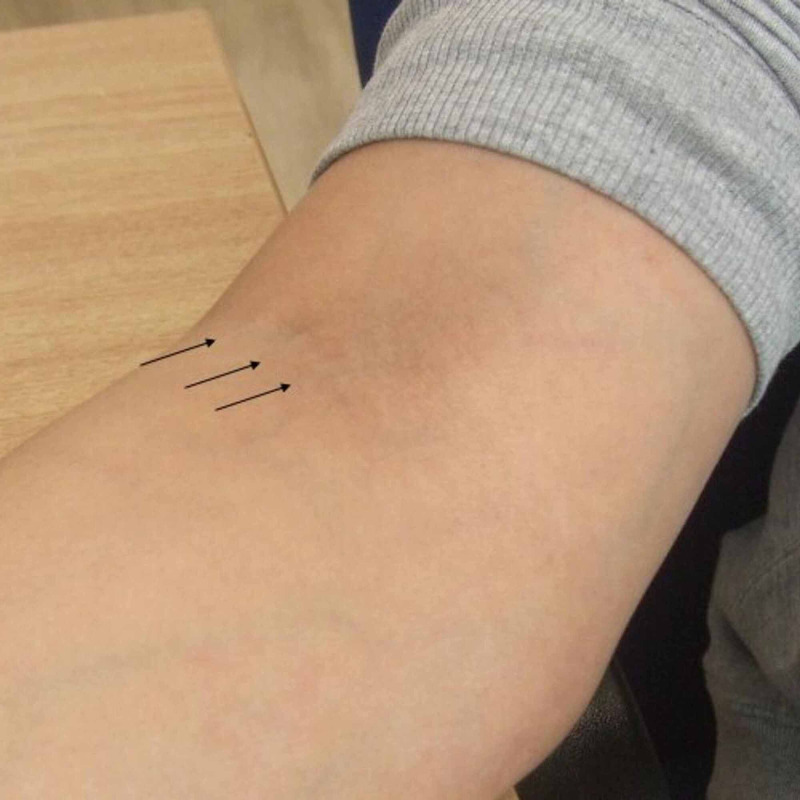
Clinical picture showing the right antecubital fossa showing cord-like bands

Her past medical history and family history were insignificant. Seven months later, she presented with painful cord-like bands in her right cubital fossa. Examination revealed three mildly tender cord-like bands which were oriented longitudinally along the length of the arm. Even though they were palpable and uncomfortable, they did not cause any restriction to the right elbow movement (Figure 1).

**Figure 2:**
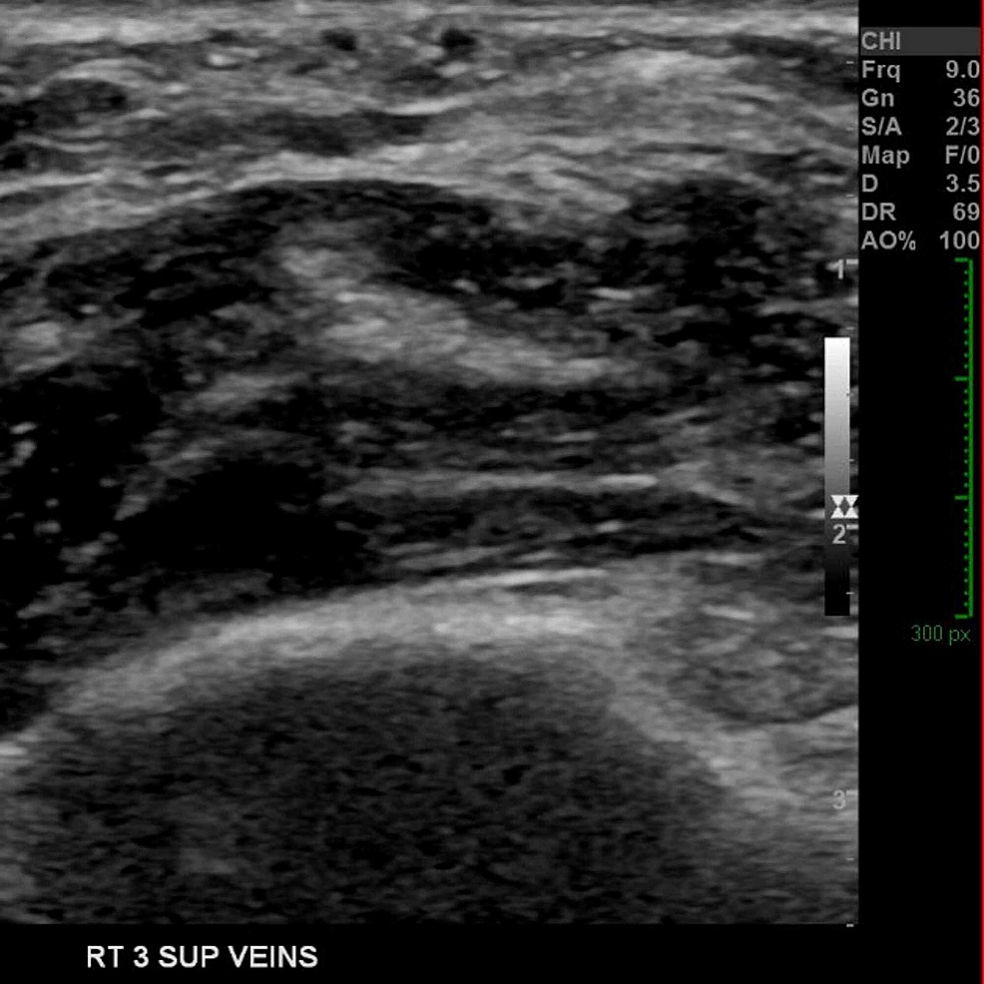
Right antecubital fossa ultrasound scan showing three linear cord-like bands (1 mm each) lying laterally within the antecubital fossa, and immediately underneath the skin.

On whole-body examination, there were no similar clinical findings in the contralateral arm or anywhere else. Right antecubital fossa ultrasound scan showed three linear cord-like bands (1 mm each) lying laterally within the antecubital fossa, immediately underneath the skin. The appearance is consistent with focal thrombus within subcutaneous veins, with no extension to cephalic or median basilica veins (Figure 2). The clinical picture was described as Mondor's disease of the arm presumably as a result of chemotherapy.

Management was conservative, which included application of nonsteroidal anti-inflammatory gel. Also, the patient was advised to perform daily digital massaging to the antecubital fossa. As there was no restriction to elbow movement or significant pain, physiotherapy was not indicated.

At the time of writing this case report (two months later), this management regime resulted in partial resolution of the fibrous bands which became softer and less painful. The patient has been advised to continue massaging the bands indefinitely. The bands have not had any negative effect on the patient’s arm function at any point.

## Discussion

Mondor's disease is a rare clinical condition with unclear aetiology. Many authors have linked it to local minor trauma, as a tight brassier, strenuous exercise or direct injury associated with surgery. In contrast, others suggested damage to the veins, hypercoagulable states, dehydration, stagnation of blood, or extrinsic pressure on veins. Also, in the past, there was a possible association between Mondor's cord and underlying breast pathology; however, no direct correlation has been proven [4].

Authors suggested that the condition starts with an inflammatory response including, pain, swelling and redness at the site leading to superficial vein fibrosis, forming a cord-like structure that can be visible and palpable. Eventually, this condition resolves itself by re-cannulisation followed by complete resolution of symptoms [3].

Mondor's disease tends to dominate in women between 30-60 years, with an incidence between 0.5% and 0.8% among breast cancer patients [5]. In most cases, Mondor's disease patients present with a painful (cord-like) structure with overlying skin erythema; ultrasound is considered the first line in the imaging showing non-compressible, hypoechoic superficial veins with absence of blood flow, and possible intraluminal thrombus [6].

As Mondor's disease is self-limiting in most cases, the natural history is for the thrombosed vein to recanalise and for clinical symptoms to resolve gradually in six to eight weeks which was the case in our case. Nonsteroidal anti-inflammatory drugs, anticoagulation therapy, triamcinolone injections and even surgery have been used for its treatment as necessary [7]. As there is always a small risk of the development of a deep venous thrombosis, adequate follow-up is recommended [8].

## Conclusions

Mondor's disease can present as an isolated superficial thrombophlebitis of the arm following chemotherapy in breast cancer patients. In most cases, it is a self-limiting condition, but supportive treatments might help to alleviate the symptoms. A close interval follow-up ultrasound scan can be useful to ensure resolution and exclude complications. Surgical intervention including excision is only required in severe cases with relief of limited joint movement.

We would recommend a metanalysis of previously reported similar cases to develop more robust data to aid diagnosis and management in order to achieve better patients' outcome.
